# Current News

**Published:** 1899-06

**Authors:** 


### Current News.

### NATIONAL DENTAL ASSOCIATION.

The annual meeting of the National Dental Association will be held in the ball-room of the International Hotel at Niagara Falls, August 1, 2, 3, and 4, 1899.

A railroad rate of one and one-third fare on the certificate plan will be obtained. Also reduced rates on C. & B. and Northern Transportation Steam-ship Lines. It is suggested that members living at a considerable distance organize parties and thereby be enabled to secure lower rates from railroad companies.

Following is a list of hotels: Cataract House, $3.00 to $4.00 per day; International Hotel, $3.00 to $4.00 per day; Kaltenback Hotel, $3.00 per day; Imperial Hotel, $2.50 to $4.00 per day; Columbia Hotel, $1.50 to $2.00 per day; Temperance House, $1.50 to $2.00 per day; Niagara Falls House, $2.00 per day; Niagara House, $2.00 per day.

Dr. M. O. Cooley, of Niagara Falls, N. Y., will engage rooms and answer any questions regarding local arrangements for the meeting. Definite meeting-places for sections will be announced later.

It is the wish of the officers of the Association that members make special efforts to be present at section meetings on account of the unusual number of valuable papers which must first be passed upon by the sections to which they properly belong.

The following is the preliminary programme:

Dr. N. S. Jenkins, Dresden, "Porcelain Enamel Inlays."

Dr. Edward H. Angle, St. Louis, "Orthodontia." (Illustrated.)

Dr. W. A. Price, Cleveland, "The Absolute Efficiency of the Controllers of the Market for Dental Cataphoresis."

Dr. L. E. Custer, Dayton, "Dental Electricity."

Dr. S. S. Stowell, Pittsfield, "The Practical Side of It."

Dr. E. P. Beadles, Danville, "A Bastard Profession."

Dr. Truman W. Brophy, Chicago, "Surgical Operations in Early Infancy for Palatal Defects."

Dr. E. K. Weldstaedt, Minneapolis, "Cements."

Dr. James Truman, Philadelphia, "The Reflexes of the Lower Molars."

Dr. J. N. Crouse, Chicago, "Operative Dentistry."

Dr. B. H. Catching, Atlanta, "Gomphosis."

Dr. R. Ottolengui, New York, "Prognathism." "Extraction and Delay *versus* Expansion and Early Attention." (Illustrated.)

Dr. W. V. B. Ames, Chicago, "Some Phases of the Cement Question."

Dr. Thomas Fillebrown, Boston, "A Study of Harelip and Cleft Palate." (Illustrated.)

Dr. M. L. Rhein, New York, "Pyorrhœa Alveolaris."

Dr. Harvey, Battle Creek, "Constitutional Deterioration the Cause of Dental Caries."

Dr. W. C. Barrett, Buffalo, "Oral Affections in Secondary Syphilis."

SUBJECTS TO BE ANNOUNCED.

Dr. W. Geo. Beers, Montreal; Dr. G. V. Black, Chicago; Dr. H. L. Ambler, Cleveland; Dr. W. H. Whistler, Cleveland; Dr. Joseph Head, Philadelphia; Dr. A. W. Harlan, Chicago; Dr. John S. Marshall, Chicago; Dr. C. N. Johnson, Chicago; Dr. A. H. Peck, Chicago; Dr. H. J. Goslee, Chicago; Dr. R. H. Hofheinz, Rochester; Dr. F. W. Low, Buffalo; Dr. G. V. I. Brown, Milwaukee; Dr. T. B. Hinman, Atlanta; Dr. H. H. Johnson, Macon; Dr. B. Holly Smith, Baltimore; Dr. C. Edmund Kells, New Orleans; Dr. M. C. Smith, Lynn; Dr. L. M. Cowardin, Richmond; Dr. Edward C. Kirk, Philadelphia; Dr. Carl Theodore Graum, Chicago; Dr. C. L. Hungerford, Kansas City; Dr. L. L. Dunbar, San Francisco; Dr. W. Ernest Walker, Pass Christian.

A revised programme, with reports from chairmen of sections, will be issued later. Prominent members of the profession from abroad have been invited to be present.

The names of the gentlemen who have promised to present papers is a sufficient guarantee of the high character of work which will be done at this meeting. The minor details will be carefully looked after and all unnecessary and irrelevant matter eliminated, so that the business of the Association may be transacted in a prompt and expeditious manner. It is hoped that the various State Societies will send full delegations, and that all members of the Association and reputable dentists in this country and Canada who are not members, will show their interest in and loyalty to the National Association by attending this meeting.

H. J. Burkhart,
*President.*
Emma Eames Chase,
*Corresponding Secretary.*
J. N. Crouse,
*Chairman Executive Committee.*

---

### AMERICAN DENTAL SOCIETY OF EUROPE.

Upon motion, the following report of the committee appointed by the American Dental Society of Europe, in London, August, 1898, was carried unanimously:

"Whereas, A Special Executive Session of the American Dental Society of Europe has been called at Brussels, April 1, 1899, for the purpose of considering what further action shall be taken towards improving the standing of the graduates from American Dental Colleges practising in Europe, and to receive and act upon the report of the Committee appointed in London at the last Annual Meeting.

"Whereas, A majority of said Committee and a large number of the active members of the Society being present from all parts of Europe, thereby showing their great interest in the subject under consideration,

"*Resolved,* That the American Dental Society of Europe views with pleasure and approval the action of the National Association of Dental Faculties, United States of America, in appointing a Foreign Relations Committee, and hopes that this Committee will be indefinitely continued, and empowered to take such action as shall appear to its members to be for the best interests of the profession.

"*Resolved,* That the Society expresses its thanks to the National Association of Dental Faculties for its resolution and action in accepting the report of its Foreign Relations Committee, and continuing it at Omaha, August, 1898, and creating Advisory Boards in all European countries, with the view that the certificates of Foreign students proposing to enter American Dental Colleges be submitted to the Advisory Boards of the respective countries of which they are citizens or residents.

"*Resolved,* That it is the opinion of this Society, that Foreign students should possess such a knowledge of the English language as will enable them to thoroughly comprehend the lectures and teachings which they will be called upon to pass examinations in, and that no foreign student should be allowed to pass any examination through the medium of an interpreter.

"*Resolved,* That the American Dental Society of Europe heartily approves of the wisdom of requiring a preliminary examination of students from European countries, or would suggest as preferable that it be required of each foreign student that he present official certificates of having passed the preliminary requirements for matriculation as a dental student in his own country, and that these certificates be endorsed by the Advisory Board of said country, and that they also be subject to the rules of the National Association of Dental Faculties.

"*Resolved,* That this Society approves of the resolution of the Foreign Relations Committee of the National Association of Dental Faculties, to appoint Advisory Boards consisting of 'not more than three members,' and it is hoped, for the accomplishment of the best results, that the number of members on each Board be raised to three at the earliest practical moment, and this Society is unanimously and strongly of the opinion that three are necessary to constitute an influential Board of this nature; and that, where practicable, at least one member should be a native of the country.

"*Resolved,* That the American Dental Society of Europe tender a sincere vote of thanks to the National Association of Dental Faculties, and to the Foreign Relations Committee, and especially to their energetic chairman, Dr. W. C. Barrett, for the active and hearty manner in which they have met the appeals of their *confrères* in Europe, who for so long have urged the importance of the consideration this subject is now receiving at their hands.

"*Resolved,* That a Committee upon Dental Education be a permanent committee of the Society, and that said committee consist of all the members of the Society who are members of the Advisory

Board of the Foreign Relations Committee of the National Association of Dental Faculties.

(Signed) "L. C. Bryan, *Chairman.*
"W. E. Royce.
"W. Mitchell."

It was further moved and carried "That the report of the Committee be sent to the various dental journals for publication at the earliest possible date."

---

### MISSOURI STATE DENTAL ASSOCIATION.

The Thirty-fifth Annual Meeting of the Missouri State Dental Association will be held in Kansas City, Mo., July 11, 12, 13, and 14, 1899. An interesting programme will be presented.

Hotel and railroad rates have been secured.

All members of the profession are cordially invited to attend.

B. L. Thorpe,
*Corresponding Secretary.*

---

### THE CHICAGO DENTAL SOCIETY—OFFICERS FOR 1899–1900.

President, Garrett Newkirk; First Vice-President, G. W. Cook; Second Vice-President, B. D. Wikoff; Secretary, Elgin Ma Whinney; Corresponding Secretary, C. S. Bigelow; Treasurer, A. B. Clark; Librarian, C. J. Merriman; Member Board of Directors, Edmund Noyes.

*Board of Censors.*—A. W. Harlan, Chairman; W. V.-B. Ames, C. N. Johnson.

C. S. Bigelow,
*Corresponding Secretary.*

100 State Street.

### NEW JERSEY DENTAL EXAMINING BOARD.

The summer meeting of the New Jersey Dental Examining Board will commence on Wednesday, July 5, at No. 88 Broad Street, Elizabeth, N. J., at ten o'clock. All preliminary credentials from applicants must be approved by the Superintendent of Public Instruction and in the hands of the secretary before June 20.

G. CARLETON BROWN,
*Secretary New Jersey Dental Examining Board.*

